# Indirect Reciprocity under Incomplete Observation

**DOI:** 10.1371/journal.pcbi.1002113

**Published:** 2011-07-28

**Authors:** Mitsuhiro Nakamura, Naoki Masuda

**Affiliations:** 1 Department of Mathematical Informatics, The University of Tokyo, Tokyo, Japan; 2 PRESTO, Japan Science and Technology Agency, Kawaguchi, Japan; Max-Planck-Institute for Evolutionary Biology, Germany

## Abstract

Indirect reciprocity, in which individuals help others with a good reputation but not those with a bad reputation, is a mechanism for cooperation in social dilemma situations when individuals do not repeatedly interact with the same partners. In a relatively large society where indirect reciprocity is relevant, individuals may not know each other's reputation even indirectly. Previous studies investigated the situations where individuals playing the game have to determine the action possibly without knowing others' reputations. Nevertheless, the possibility that observers of the game, who generate the reputation of the interacting players, assign reputations without complete information about them has been neglected. Because an individual acts as an interacting player and as an observer on different occasions if indirect reciprocity is endogenously sustained in a society, the incompleteness of information may affect either role. We examine the game of indirect reciprocity when the reputations of players are not necessarily known to observers and to interacting players. We find that the trustful discriminator, which cooperates with good and unknown players and defects against bad players, realizes cooperative societies under seven social norms. Among the seven social norms, three of the four suspicious norms under which cooperation (defection) to unknown players leads to a good (bad) reputation enable cooperation down to a relatively small observation probability. In contrast, the three trustful norms under which both cooperation and defection to unknown players lead to a good reputation are relatively efficient.

## Author Summary

Humans and other animals often help others even when the helping behavior is costly. Several mechanisms can explain the emergence and maintenance of cooperation. In one such mechanism called indirect reciprocity, individuals are rated according to their past behavior toward others. Individuals help others with a good reputation but not those with a bad reputation. Indirect reciprocity is relevant in relatively large societies where individuals do not meet each other repeatedly. Then, unless an authority maintains the reputation of individuals, individuals would not know information about some others even indirectly via gossip. We investigated a model in which both individuals playing the game (acting players) and observers of the game, who evaluate acting players and start gossiping, incompletely perceive others. In the unique viable and cooperative strategy, one cooperates with good and unknown peers and defects against bad peers. Populations of suspicious observers under which cooperation (defection) to unknown peers is regarded to be good (bad) enable cooperation in relatively wide parameter regions. In contrast, populations of trustful observers under which both cooperation and defection to unknown peers are regarded to be good are relatively efficient.

## Introduction

We often help others even when the helping behavior is costly. The Prisoner's Dilemma game and its variants are used for examining cooperative behavior in such social dilemma situations. Several mechanisms can explain the emergence and maintenance of cooperation [1], [2]. Direct reciprocity, one such mechanism, is relevant when the same pair of players repeatedly interact [3], [4]. To avoid retaliation from a peer player, it is beneficial for both players to maintain cooperation. However, direct reciprocity cannot explain cooperation in relatively large societies, where players do not repeatedly meet each other.

Indirect reciprocity is a main mechanism for cooperation in cases where players never interact with the same partners [5]–[8]. In indirect reciprocity, players are motivated to help others and receive help from different others. There are two types of indirect reciprocity mechanisms: upstream and downstream reciprocity [2],[9],[10]. The two types of indirect reciprocity differ in the direction of the chain of helping behavior. In upstream reciprocity, a player is motivated to help after the player has been helped by someone. In downstream reciprocity, a player will be helped after the player has helped someone. In downstream reciprocity, players possess unique reputation scores, determined by their past actions toward other players. Players help others with a good reputation but not those with a bad reputation. Nowak and Sigmund proposed a computational model in which players helping others are regarded to be good and those withdrawing help are regarded to be bad [6], [7]. According to their model, helping others to maintain a good reputation is more beneficial than withdrawing help to gain momentary profits. Empirical studies also support downstream reciprocity [11]–[14]. In the present study, we focus on downstream reciprocity and simply refer to it as indirect reciprocity.

The rule according to which players decide either to cooperate or to defect based on the reputations of the relevant players is called the action rule [15], [16]. The discriminator that helps those with a good reputation and does not help those with a bad reputation is an example of the action rule. The rule for assigning a reputation to players based on their actions is called the social norm [15], [16]. Nowak and Sigmund's norm is termed image scoring [6], [7]. Theoretically, the discriminator is an unstable action rule under image scoring because the discriminator is invaded by the unconditional cooperator [17]–[19]. The discriminator is stable under some more complex social norms including standing [15], [17], [18], [20]–[23], judging [15], [21]–[24], and shunning [10], [23], [25]. These complex social norms require more information about other players than image scoring does, such as the co-player's reputation, in addition to the information on the player's action toward the co-player.

Unless an authority maintains the reputation of all the players, as in the case of online marketplaces [26], [27] and communities of medieval merchants [28], the information about players, which is indispensable for indirect reciprocity, must spread from players to players via gossiping [10], [14], [15], [29]. However, except in a sufficiently small population, the accuracy and span of gossip may be limited [16], [30], [31]. In such a case, the information about players is shared incompletely in the population, and individuals often need to make decisions when the information about the relevant players is unknown.

**Figure 1:**
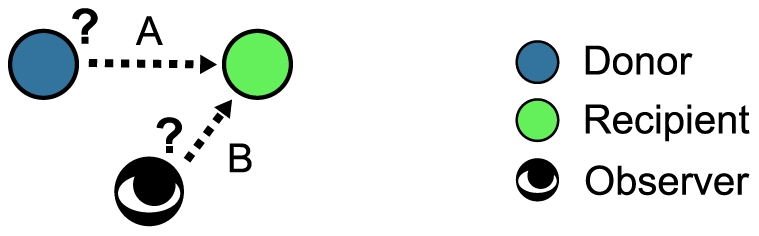
Two types of incomplete observation in a one-shot game. We distinguish two types of observation. First, an interacting player (actually, donor) knows or does not know the co-player's (actually, recipient's) reputation (A). Second, an observer of the game knows or does not know the co-player's reputation (B). Previous studies have treated only the incompleteness of type A.

Several studies have addressed the case in which players do not necessarily know the reputation of others [6], [7], [18], [19], [21], [30], [31]. However, these studies have two limitations. First, it is assumed in these studies that only players playing the game, not the third-party observers of the game, incompletely perceive the reputation of other players. The role of the third-party observer is to generate the reputation about players and disseminate it to other players in the population. The observer can propagate the reputation about players to others only when the observer knows the reputation about the players in question. Figure 1 illustrates the point. In a one-shot game, a player knows or does not know the co-player's reputation (A). In addition, an observer, who watches the game but does not play the game, knows or does not know the co-player's reputation (B). Previous studies considered incomplete observation of type A but not B. In reality, however, the interacting player and the observer are roles that the same individual may play on different occasions such that both roles may accompany incomplete access to information about others.

Second, these studies examined the sustainability of a few exemplary combinations of the social norm and action rule (e.g., combinations of the image scoring norm and the discriminator action rule [6]). The choice of the pairs of social norm and action rule is subjective. On the other hand, exhaustive studies in which all the pairs of social norm and action rule within a certain class are examined are not concerned with the issue of incomplete information [15]. These studies considered erroneous behavior, such as wrong assignment of the reputation to other players. However, the error probability is eventually set to be infinitesimally small. We assume that the information about the reputation is available to interacting players and observers with an arbitrary probability between 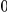 and 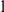.

In the present study, we perform an exhaustive search to explore the possibility of indirect reciprocity under the social norms that permit observers to assign reputations to unknown players. The manner in which individuals may know the reputation about others generally depends on details of information spreading processes (e.g., gossiping on a social network). We do not consider explicit mechanisms of information spreading and model the incomplete observation by the probability with which a player and an observer know the reputation of the co-player in a one-shot game. In particular, we investigate two types of observation: concomitant and independent observation (see Results). Our exhaustive analysis reveals that the trustful discriminator that helps players with a good or unknown reputation and does not help players with a bad reputation is the only self-supporting action rule under several social norms. Even if the fraction of players knowing others' reputation is relatively small, the population can be sufficiently cooperative.

## Methods

### Model

We generalize the model of indirect reciprocity derived from the donation game with binary reputation values [7], [15], [16], [18], [19], [21]–[23], [25], [30]–[32] with an additional assumption that players know the reputation of a fraction of other players. Consider an infinitely large population. From this population, we arbitrarily select two players, one as a donor and the other as a recipient. The donor either cooperates (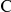) with or defects (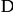) against the recipient. If the donor cooperates, the donor pays cost 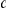, and the recipient gains benefit 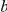. We assume 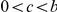 such that the defection is rational for the donor in a one-shot game, whereas cooperation contributes to the welfare of the population. We repeat the same procedure until each player is paired with a sufficient number of others but never with the same opponent. In this way, we exclude direct reciprocity. Consequently, the participation of each player in the games as a donor and a recipient is equally probable.

Each player possesses a binary reputation value, i.e., good (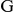) or bad (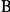). We assume that a third player serves as an observer of a one-shot game and assigns 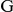 or 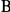 to the donor. In a one-shot game, the donor and the observer know the reputation of the recipient with probability 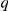 (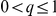). The probability that the reputation of the recipient, which is actually 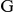 or 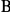, is unknown (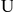) to the donor and the observer is 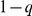. The recognition of the reputation by the donor and that by the observer are assumed to occur concomitantly or independently (see Results). In contrast to previous studies, observers as well as donors in our model are imperfect with regard to knowing the recipients' reputation.

The donor's action (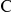 or 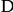) depends on the recipient's reputation (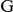, 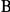, or 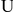 in the donor's eyes). For example, a player that cooperates with a 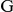 recipient, represented as 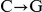, and also cooperates with 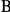 and 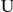 recipients (i.e., 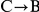 and 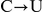) is referred to as the unconditional cooperator (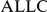). A player obeying the action rule 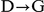, 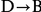, and 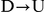 is called the unconditional defector (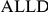). A player obeying the action rule 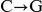, 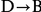, and 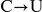 is a discriminator that also cooperates with recipients whose reputation is unknown to the donor; this discriminator is denoted by 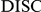. Because an action rule is specified by the allocations of 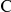 or 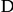 to each of 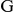, 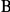, and 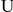, there are 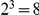 action rules.

**Figure 2:**
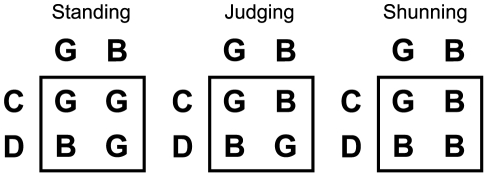
Second-order social norms when 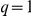. Second-order social norms that realize indirect reciprocity when 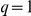. They are termed simple standing, stern judging, and shunning. In this paper, we simply refer to them as standing, judging, and shunning, respectively. Under these social norms, the discriminator is stable and cooperative.

The observer updates the reputation of the donor based on the donor's action (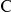 or 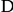) and the recipient's reputation (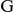, 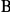, or 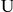 in the observer's eyes). We refer to the update rule as the social norm. The class of social norms that we are considering is called the second-order assessment [10]; the update rule depends on two kinds of information: the donor's action and the recipient's reputation. When 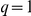 (therefore, no 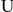 recipients), simple standing, stern judging, and shunning, which are schematically shown in Fig. 2, belong to this class. To simplify notation, we henceforth refer to simple standing, stern judging, and shunning as standing, judging, and shunning, respectively. For example, in the case of standing, the observer assigns reputation 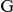 when the donor cooperates with a good recipient (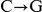) or when the recipient is bad (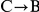, 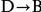) and assigns 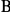 when 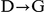. Because a second-order social norm is specified by the allocations of 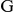 or 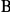 to each of 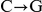, 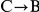, 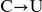, 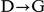, 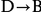, and 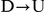 when 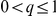, there are 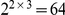 social norms.

We also assume that the donor receives a new reputation opposite to that intended by the observer with a small probability 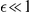. With probability 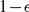, the observer assigns a reputation to the donor according to the social norm. This error models the limited ability of the observer. Another reason for introducing the error is that 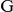 and 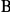 players must coexist in the population for distinguishing the efficiency of different social norms and action rules.

### Analysis Methods

We analyze the stability and cooperativeness of the homogeneous population of each of the 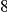 action rules under each of the 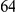 social norms by adopting the exhaustive search method introduced in Refs. [15], [16]. Given a value of 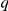, we check whether each combination of the social norm and action rule (there are 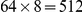 combinations in total) satisfies the following two properties.

**Stability:** For a given social norm, an action rule is a strict Nash equilibrium (NE), if the payoff of the action rule against itself is greater than the payoff of any other action rule against the focal action rule.

**Cooperativeness:** For a given social norm, an action rule is cooperative, if players in the homogeneous population in which everyone adopts the focused action rule cooperate with a sufficiently large probability.

The precise procedure is as follows.

#### Stability

For a social norm and a value of 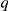, consider an almost homogeneous population in which almost all the players adopt action rule 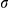 and an infinitesimal fraction of mutant players adopt action rule 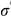. We examine the stability of 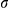 against 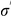. We denote by 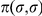 and 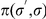 the payoffs that players obeying 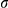 and 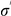, respectively, obtain in the almost homogeneous population of players obeying 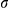. Note that the payoff is defined as the expectation of the accumulated payoff obtained by playing many one-shot games. We assume that the number of the games that each player plays is fixed and sufficiently large and that a player is not paired with the same partner more than once. We examine the strong Nash stability using 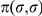 and 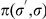; we are not concerned with population dynamics. An action rule 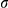 is a strict NE if 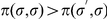 for all the other 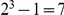 action rules 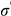 (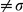). If 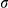 is a strict NE, 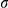 is also an evolutionarily stable strategy (ESS).

Let 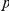 be the probability that the reputation of a player in the homogeneous population of players obeying 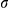 is equal to 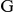. After a sufficient number of games, 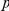 relaxes to the unique stable equilibrium 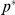 [15] determined by
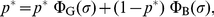
(1)where 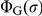 and 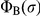 are the probabilities that the reputation of a donor obeying 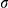 becomes 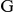, given that the recipient has reputation 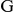 and 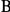, respectively. After a single-shot game, a donor's reputation may become 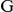 via either of the following two events. First, the donor may meet a 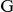 recipient with probability 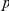, and the donor's action toward the recipient, in accordance with action rule 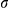, is regarded to be 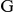 with probability 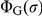. Second, the donor may meet a 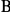 recipient with probability 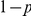, and the donor's action toward the recipient is regarded to be 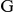 with probability 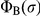. The two terms on the right-hand side of Eq. (1) represent the probabilities of the two events in the equilibrium. 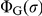 and 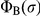 depend on the specificity of how donors and observers know the recipients' reputation and are described in Results. For example, a social norm that regards any action of donors (i.e., 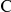 or 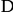) to be 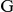 gives 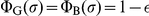.

If a small number of mutants obeying 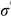 exist in the almost homogeneous population of players obeying 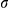, the probability that a mutant has reputation 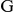, denoted by 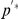, is determined by
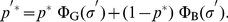
(2)In the equilibrium, almost all the players obey 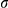, and they have 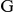 reputation with probability 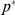. Then, a mutant donor obeying 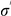 may meet a 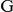 recipient with probability 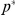, and the donor's action toward the recipient is regarded to be 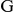 with probability 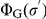. Alternatively, the mutant donor may meet a 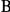 recipient with probability 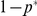, and the donor's action toward the recipient is regarded to be 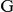 with probability 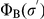. The two terms on the right-hand side of Eq. (2) represent the probabilities of the two events. The right-hand side of Eq. (2) can be calculated by using 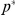 obtained by solving Eq. (1).

A donor obeying action rule 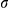 cooperates in one of the following three ways. First, the donor may sense the recipient's reputation with probability 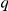, the recipient's reputation is 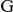 with probability 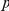, and the donor cooperates if the donor is supposed to cooperate with 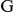 recipients under action rule 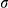. Second, the donor may sense the recipient's reputation with probability 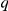, the recipient's reputation is 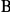 with probability 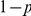, and the donor cooperates if the donor is supposed to cooperate with 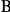 recipients under action rule 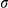. Third, the donor does not sense the recipient's reputation with probability 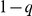 and cooperates if the donor is supposed to cooperate with 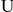 recipients. Therefore, the probability that a donor obeying 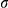 cooperates, 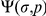, is given by
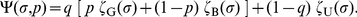
(3)

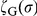
, 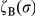, or 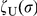 is equal to 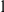 when the donor obeying 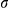 cooperates with the recipient having reputation 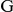, 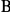, or 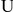, respectively. Otherwise, 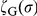, 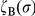, or 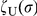 is equal to 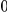. For example, 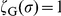, 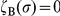, and 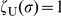 if 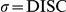.

The expected payoffs in a single donation game in the equilibrium are given by
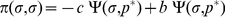
(4)and
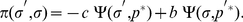
(5)The first terms on the right-hand side of Eqs. (4) and (5) represent the cost when the player is a donor and the second terms represent the benefit when the player is a recipient. We have neglected the proportionality constant 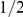.

#### Cooperativeness

A strict NE action rule may not be sufficiently cooperative. 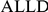 is such an example. To exclude non-cooperative equilibria, we use the criterion of cooperativeness introduced in Refs. [15], [16]. We expand 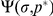 in a power series in terms of the probability of assignment error 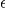 as
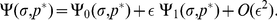
(6)Action rule 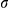 is defined to be cooperative if 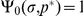. In this case, the player always cooperates as donor in the limit of no assignment error. For example, consider a homogeneous population of players adopting 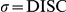 under a social norm that regards any action of the donor (i.e., 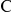 or 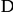) to be 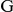, except for the assignment error. Then, we obtain the obvious steady state of the reputation 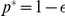. By substituting 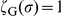, 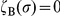, and 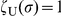, which describes 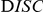, and 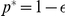 in Eq. (3), we obtain 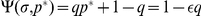. Therefore, 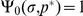 and 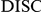 satisfies the condition of cooperativeness under this social norm. On the other hand, in a homogeneous population of players adopting 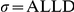, 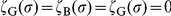 so that 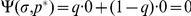. Therefore, 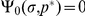. 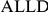 does not satisfy the condition of cooperativeness under any social norm.

#### Summary of the Methods

In summary, we check the stability and cooperativeness of action rule 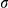 under a given social norm, the values of 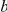, 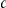, and 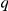, as follows:

1. Calculate 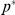 from Eq. (1).
2. Calculate 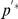 by substituting 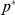 in Eq. (2).
3. Calculate 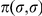 by substituting 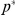 in Eqs. (3) and (4).
4. For each of the other seven action rules 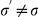,
  - Calculate 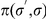 by substituting 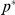 and 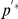 in Eqs. (3) and (5).
  - If 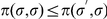, 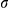 is unstable against 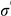.
5. If 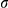 is stable against all the seven action rules 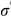,
  - Calculate 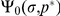 using Eqs. (3) and (6).

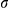
 is cooperative if 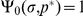.

## Results

**Figure 3:**
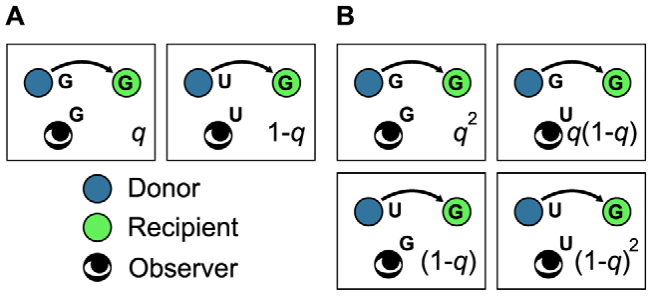
Different patterns of observation. Different patterns of observation of the recipient's reputation. (A) Concomitant observation. Both the donor and observer know the recipient's reputation with probability 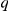, and neither of them knows the recipient's reputation with probability 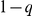. (B) Independent observation. Both the donor and observer know the recipient's reputation with probability 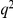, only the donor knows the recipient's reputation with probability 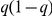, only the observer knows the recipient's reputation with probability 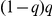, and neither of them knows the recipient's reputation with probability 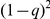.

We deal with two types of observation. Subsection “Concomitant Observation” is devoted to the analysis of the so-called concomitant observation (Fig. 3(A)). The subsequent three subsections are devoted to the so-called independent observation (Fig. 3(B)).

### Concomitant Observation

In this section, we assume that the recipient's reputation in a single game is known or not known by the donor and the observer concomitantly. There are two possible situations in a single game (see Fig. 3(A)). With probability 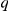, both the donor and observer know the recipient's reputation. With probability 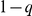, neither the donor nor the observer knows the recipient's reputation. 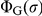 and 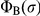, used in Eqs. (1) and (2), are given by
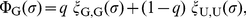
(7)

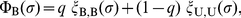
(8)where 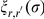 is the probability that the action of the donor obeying 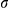 is regarded to be 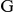 by the observer, 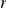 is the recipient's reputation in the donor's eyes, and 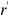 is the recipient's reputation in the observer's eyes. Note that 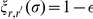 and 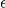 if the donor's action is regarded to be 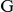 and 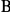 except in the case of the assignment error, respectively.

We found that, except for 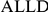, there are 24 pairs of social norms and action rules in which the action rule is a strict NE. The number of pairs should actually be considered as 12 because the system is invariant if we flip 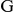 and 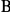 in all the entries of the social norm and the action rule [15]. For example, consider the following two pairs X and Y of social norm and action rule. X consists of the social norm under which donors always receive 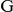 and the action rule 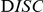, i.e., 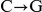, 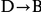, and 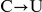. Y consists of the social norm under which donors always receive 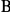 and the action rule 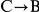, 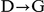, and 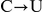. Because we obtain Y by flipping 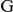 and 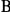 in X, X and Y are essentially the same. Among the 12 strict NE pairs, three pairs are cooperative. The unique action rule that is cooperative under each of the three social norms is 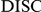.

**Figure 4:**
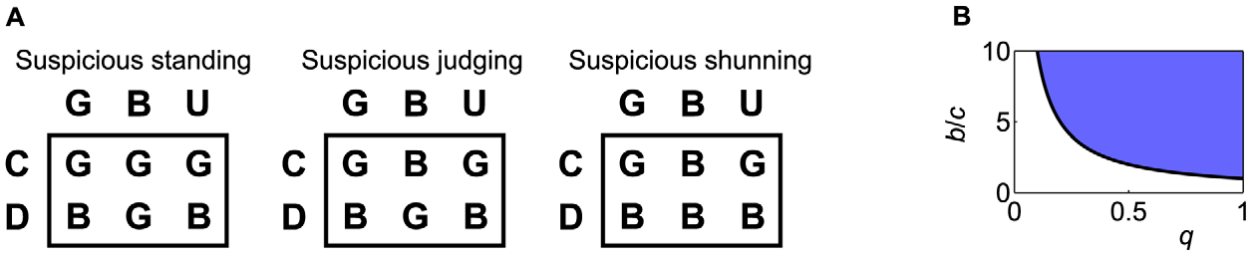
Social norms (concomitant observation). Social norms that realize indirect reciprocity in the case of concomitant observation. (A) Suspicious standing, suspicious judging, and suspicious shunning. (B) Under these three social norms, 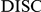 is stable and cooperative in the shaded parameter region, which is given by Eq. (9). The bold line represents 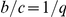.

The three social norms are schematically shown in Fig. 3, where rows represent the donor's actions and columns represent the recipient's reputations. They are common in that the cooperation with a 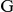 or 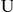 recipient is regarded to be 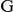 and the defection against a 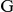 or 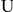 recipient is regarded to be 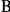. Under these social norms, observers *suspect* that donors defecting against 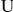 recipients are defectors. Therefore, we refer to these social norms as suspicious social norms, namely, suspicious standing, suspicious judging, and suspicious shunning (Fig. 4(A)). The suspicious social norms generalize standing, judging, and shunning, which are the unique stable and cooperative second-order social norms when everybody knows the reputation of each other (i.e., 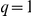; Fig. 2) [23].

Under all the three social norms, 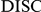 is stable in the shaded parameter region in Fig. 4(B), i.e.,
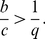
(9)Equation (9) is also required for indirect reciprocity in a model with a different assumption for incomplete observation of reputations [6],[7],[10]. Generally speaking, the probability 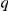 of knowing the reputation of others must be greater than the cost-to-benefit ratio 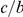 for sustaining indirect reciprocity. When 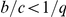, 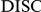 is invaded by six action rules, i.e., all the other action rules except 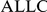.

### Independent Observation

In this section, we assume that the recipient's reputation in a single game is known or not known by the donor and the observer independently. There are four possible situations in a single game (see Fig. 3(B)). First, both the donor and observer know the recipient's reputation, with probability 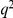. Second, only the donor knows the recipient's reputation, with probability 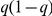. Third, only the observer knows the recipient's reputation, with probability 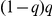. Finally, neither of them knows the recipient's reputation, with probability 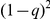. 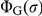 and 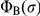, used in Eqs. (1) and (2), are given by
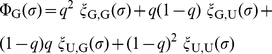
(10)and
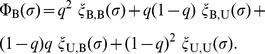
(11)


**Figure 5:**
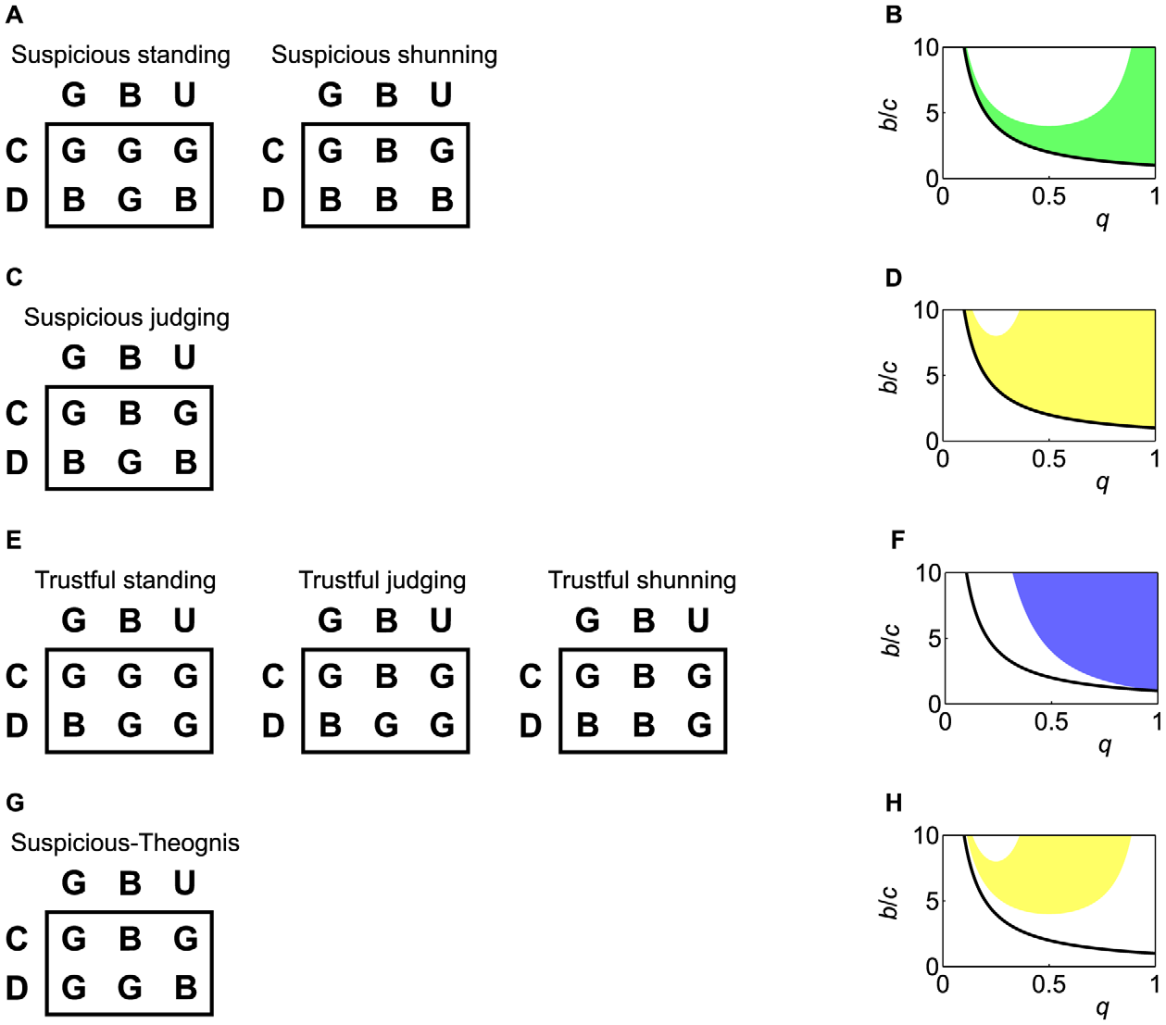
Social norms (independent observation). Social norms that realize indirect reciprocity in the case of independent observation. (A) Suspicious standing and suspicious shunning. Under these two social norms, 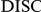 is stable and cooperative in the shaded parameter region in (B), which is given by Eq. (12). (C) Suspicious judging. Under this social norm, 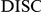 is stable and cooperative in the shaded parameter region in (D), which is given by Eq. (13). (E) Trustful standing, trustful judging, and trustful shunning. Under these three social norms, 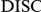 is stable and cooperative in the shaded parameter region in (F), which is given by Eq. (14). (G) Suspicious-Theognis. Under this social norm, 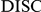 is stable and cooperative in the shaded parameter region in (H), which is given by Eq. (15). The bold lines in (B), (D), (F), and (H) represent 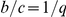.

We found that, except for 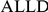, there are essentially 27 pairs of social norms and action rules in which the action rule is a strict NE. Seven of these 27 pairs are cooperative. As in the case of the concomitant observation (see subsection “Concomitant Observation” above), the unique action rule that is cooperative under each of the seven social norms is 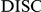. Figure 5(A), 5(C), 5(E), and 5(G) represents the seven social norms. The corresponding parameter regions in which 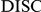 is stable under these social norms are shown in Fig. 5(B), 5(D), 5(F), and 5(H).

The three social norms shown in Fig. 5(A) and 5(C) are those found in the case of the concomitant observation (Fig. 4(A)), i.e., suspicious standing, suspicious judging, and suspicious shunning. Under suspicious standing and suspicious shunning (Fig. 5(A)), 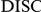 is stable in the shaded parameter region in Fig. 5(B), i.e.,
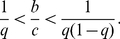
(12)Under suspicious judging (Fig. 5(C)), 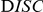 is stable in the shaded parameter region in Fig. 5(D), i.e.,
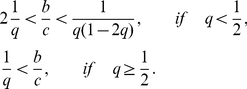
(13)The condition 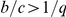 is similar to that for the concomitant observation (Eq. (9)). When 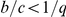, 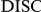 is invaded by six action rules, i.e., all the other action rules except 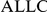. In contrast to the case of the concomitant observation, there are upper bounds of 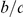 for 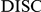 to be stable under the three suspicious social norms. When 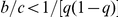 in Eq. (12) or 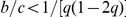 in Eq. (13) is violated, 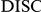 is invaded by 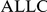 for the following intuitive reason. Because of the probability 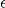 (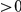) with which the assignment error occurs, the reputation of some 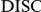 players is 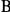. Let us suppose that a recipient's actual reputation 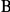 is correctly known by the donor but not by the observer; the recipient's reputation in the observer's eyes is 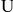. This event can occur in the case of the independent, but not concomitant, observation. In this situation, a 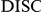 donor 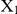 defects against the recipient and gains a 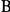 reputation. Meanwhile, an 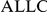 donor 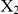 cooperates and gains a 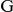 reputation. Then, 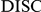 donors in later rounds help 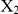 but not 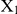. Therefore, 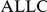 invades 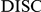.

The three social norms shown in Fig. 5(E) constitute another set of generalizations of standing, judging, and shunning. They differ from the suspicious social norms (Figs. 4(A), 5(A), and 5(C)) in that the defection against a recipient having reputation 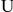 in the observer's eyes is regarded to be 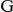. Under these social norms, observers *trust* donors defecting against 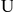 recipients by supposing that the donors are discriminators defecting against 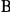 recipients and not that the donors are mere defectors. Therefore, we call them trustful social norms, i.e., trustful standing, trustful judging, and trustful shunning. Under the three trustful social norms, 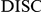 is stable when
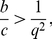
(14)which is a stricter condition than 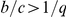. 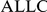 does not invade 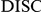 under these trustful social norms. Intuitively, this is because defection against a 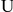 recipient in the eyes of the observer is regarded to be 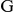, which cancels the superiority of 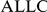 over 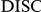 that is present under the suspicious social norms. However, 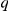 must be larger than that in the case of the suspicious social norms to prevent invasion by other action rules. This is because observers do not assign a 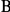 reputation and cannot discriminate mere defectors from discriminators when the recipient's reputation is 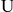 in the observer's eyes. When 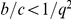, 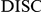 is invaded by six action rules, i.e., all the other action rules except 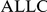.

The social norm shown in Fig. 5(G) is not a variant of standing, judging, or shunning. Because cooperation with 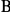 recipients is only regarded to be 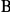 when the recipient's reputation is known under this social norm, we name this social norm suspicious-Theognis after the ancient Greek poet Theognis of Megara, who said “He that doeth good to the baser sort suffereth two ills—deprivation of goods and no thanks” [33]. Suspicious-Theognis is the same as the suspicious judging (Fig. 5(C)) except that under suspicious-Theognis, defection against 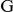 recipients in the eyes of the observer is regarded to be 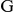. This assignment event can occur only when the recipient actually has a 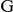 reputation. In this situation, the 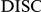 donor never defects; the 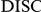 donor defects only when the recipient actually has 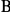 reputation. Consequently, the 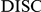 player's payoff is the same under suspicious judging and suspicious-Theognis, whereas the parameter region in which 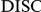 is stable against the other action rules differs for the two social norms.

Under suspicious-Theognis, 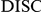 is stable in the shaded parameter region in Fig. 5(H), i.e.,
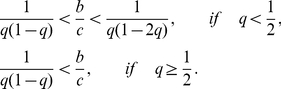
(15)The condition 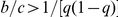 is severer than 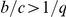, which corresponds to suspicious judging (Eq. (13)). Regardless of the value of 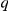, 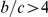 is necessary for cooperation under suspicious-Theognis (Eq. (15)); however, as 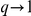, only 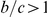 is needed under the other six social norms including suspicious judging. When 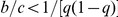, 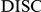 is invaded by six action rules, i.e., all the other action rules except 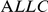. If 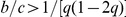, 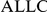 invades 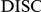 for the same reason as that for the three suspicious social norms shown in Fig. 5(A) and 5(C). Paradoxically, the condition under which 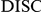 is stable is severe when 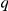 is large. When observers know recipients' reputations, they always assign 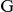 to donors defecting against recipients. Therefore, when 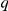 is large, 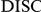 is invaded by other defective action rules. In the limit 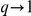, 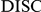 is unstable regardless of the value of 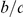. In contrast, the other six social norms shown in Fig. 5(A), 5(C), and 5(E) converge to the conventional standing, judging, or shunning norms in the limit 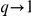. Our results obtained in this and the previous sections are consistent with those in the previous literature obtained for 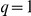 [23].

### Different Probabilities of Knowing the Recipient's Reputation by Donor and Observer under Incomplete Observation

In Model, we assumed that donors and observers know the reputation of recipients with the same probability 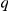. However, this probability may also be different for donors and observers because a player may have different interests or attention levels depending on whether the player faces a game as donor or observer. In the case of concomitant observation (see Results), this distinction is irrelevant. Let 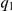 and 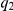 be the probabilities that the donor and the observer know the recipient's reputation in a single game, respectively. In the case of independent observation, the parameter regions in which 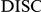 is stable are shown in Table 1.

**Table 1:** Stability regions for DISC when donors and observers may have different amount of information.

Social norms	Parameter regions in which 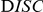 is stable
Fig. 5(A)	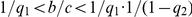
Fig. 5(C)	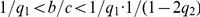
Fig. 5(E)	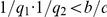
Fig. 5(G)	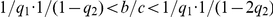

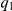
 and 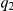 are the probabilities that the donor and the observer know the recipient's reputation in a single game, respectively. If the benefit-to-cost ratio 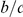 is smaller than the lower bound, 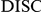 is invaded by six action rules, i.e., all the other action rules except 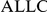. If 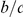 is larger than the upper bound, 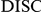 is invaded by 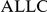. To prevent the invasion by the six action rules, 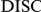 donors must have sufficient information about recipients' reputations under all the social norms. To prevent the invasion by 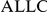, observers must have sufficient information about recipients' reputations under suspicious social norms (Fig. 5(A), Fig. 5(C), and Fig. 5(G)). This is not the case under trustful social norms (Fig. 5(E)).

Table 1 indicates that all the four conditions contain the factor 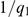 in their lower bounds of 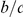. This implies that if donors know recipients' reputation with a large probability, 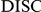 is relatively resistant to invasion by six action rules, i.e., all the other action rules except 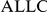.

Three of the four conditions (except for the trustful social norms shown in Fig. 5(E)) have upper bounds of 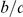 that also contain the factor 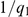. Therefore, if donors know recipients' reputations sufficiently frequently, 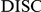 is invaded by 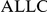. The reason for this is the same as that described in subsection “Independent Observation” above. 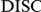 donors defect against recipients if they know that the recipients' reputations are 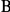, whereas such defection is regarded to be 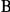 if the observers do not know the recipients' reputations. In contrast, 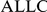 donors do not receive 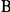 reputation via this route. However, because the three upper bounds of 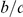 contain the factor 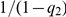 or 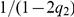, a large value of 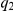 prevents the invasion by 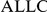. This is because, if the observers know the recipients' reputations sufficiently frequently, 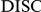 donors' defection against 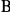 recipients is judged as 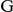. As explained in “Independent Observation”, the situation in which the donor does and the observer does not know the recipient's reputation crucially affects the upper bounds of the parameter region in which 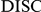 is stable. The lower bound of 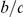 for suspicious-Theognis (Fig. 5(G)) contains the factor 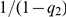. Under this social norm, the blindness of the observer enlarges the stability region of 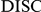. This occurs intuitively because if observers know recipients' reputation with a large probability, defection tends to be regarded as 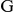.

### Comparison of Different Social Norms under Incomplete Observation

To identify the most efficient of the seven social norms, we compare them in terms of the payoff that the 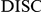 player obtains. In the homogeneous population, the payoff of 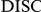 is given by 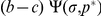. Therefore, the question of highest efficiency is reduced to the comparison of 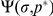 derived from the different social norms. In Eq. (6), 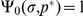 is satisfied under all the seven social norms because we have imposed cooperativeness. Thus, we compare 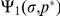 in Eq. (6). Because the payoff of 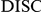 under the suspicious judging and suspicious-Theognis norms is exactly the same, we compare the payoffs of 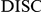 under the six social norms shown in Fig. 5(A), 5(C), and 5(E).

**Figure 6:**
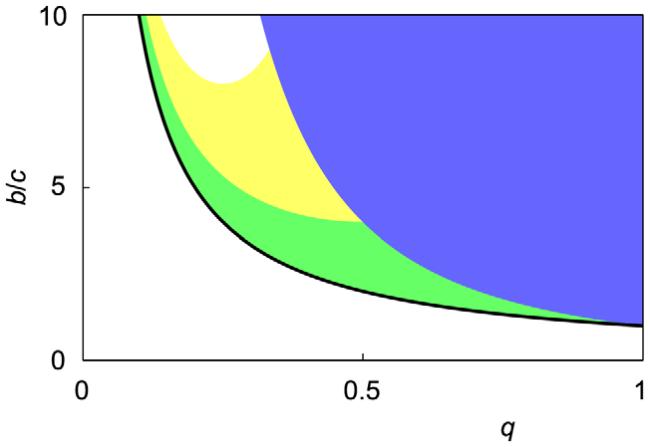
Most efficient social norms. Social norms under which 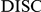 is the most efficient. In the blue region, trustful standing is the most efficient. In the green region, suspicious standing is the most efficient. In the yellow region, suspicious judging and suspicious-Theognis are equally the most efficient. Outside these regions, only 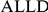 is stable. The bold line represents 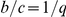.

Figure 6 shows the social norms that realize the largest payoff of 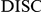 for various values of 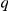 and 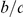. Trustful standing is the most efficient when
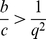
(16)holds (blue region). Suspicious standing is the most efficient when
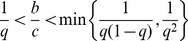
(17)holds (green region). These two social norms are variants of standing. 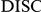 under the suspicious judging and suspicious-Theognis has an equal and the highest payoff when
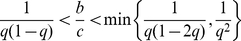
(18)holds (yellow region). This parameter region (yellow) is narrower than those in which the variants of standing are the most efficient (blue and green). Nowhere in the parameter region are variants of shunning the most efficient. When 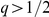, only the variants of standing are the most efficient. When 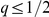, the variants of standing and judging are the most efficient for different ranges of 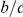 and 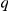. Variants of standing are the most efficient in a broad parameter region; this is intuitively because observers under variants of standing assign 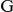 to donors more often than observers under variants of judging and shunning and because the fraction of cooperation increases with the number of 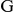 players. However, to prevent the invasion by defectors, observers should assign 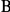 to inappropriate donors.

## Discussion

The present study is motivated by the premise that in a relatively large-scale society, players and observers may not know each other even indirectly. Under any viable social norm, the unique action rule 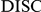 stabilizes a cooperative society. 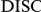 cooperates with good and unknown recipients and defects against bad recipients. 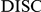 behaves trustfully toward (i.e., cooperates with) unknown recipients, and such a trustful discriminator also supports cooperation in other models of indirect reciprocity [6], [7], [18], [19], [31], [32]. We emphasize that we did not prefabricate 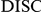 but derived it through an exhaustive search.

Previous studies only focused on social norms of discrete orders. Under first-order social norms (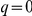 for observers), observers have no information about the reputation of players. Under higher-order social norms (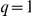 for observers), observers have the complete information about the reputation of players. We set 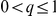 for observers as well as for donors. The social norms that we discovered can be classified into suspicious social norms in which observers discriminate between cooperative and defective donors interacting with unknown recipients and trustful social norms in which observers always assign a good reputation to donors interacting with unknown recipients. In the case of independent observation, there is a trade-off between trustful and suspicious social norms. Trustful social norms are more efficient in the sense that they yield the highest payoff of 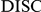 when they are stable (blue region in Fig. 6), while suspicious social norms enable indirect reciprocity down to a smaller value of 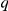. We have only considered the case in which all the players in a population obey a unique social norm. Note that a few recent studies investigated competition between players obeying different norms [34]–[36]. In contrast, such a trade-off does not exist for donors; trustful donors are always better than suspicious donors in our model and in the previous models [2], [7], [18].

The exhaustive search method was pioneered by Ohtsuki & Iwasa [15]. In Ref. [15], the combinations of third-order social norm and action rule under complete observation are exhaustively searched. By definition, the third-order social norms and action rules depend not only on the donor's action and the recipient's reputation but also on the donor's reputation. Ohtsuki & Iwasa [15] found that the eight third-order social norms, called the leading eight, sustain indirect reciprocity. The discriminator or the so-called contrite TFT is stable and cooperative depending on the social norm included in the leading eight. The leading eight possesses properties similar to those of the stable and cooperative second-order social norms that we discovered. The leading eight includes essentially second-order simple-standing and stern-judging, whose extensions were identified as stable and cooperative social norms in the present study. In contrast, shunning, which we discovered in the extended form, is not included in the leading eight. This discrepancy is caused by the different assumptions regarding incomplete observation employed in these studies; Ohtsuki & Iwasa set 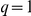, and we set 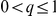. If 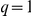, observers obeying shunning always assign 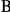 to donors when recipients have 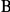 reputation. Therefore, the reputation dynamics leads to a large fraction of 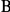 players. If 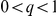, observers may assign 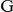 to donors when the observers do not know the recipients' reputations. In fact, the results for shunning are qualitatively different between the cases 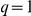 and 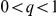. We did not explore third-order social norms (i.e., social norms using donors' reputations) with incomplete observation (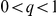) because it would be difficult to comprehend plethora of results obtained from the exhaustive search of third-order social norms with 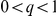. Instead, we found that the trustful and suspicious second-order social norms, which are distinguished for 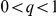, sustain indirect reciprocity.

**Figure 7:**
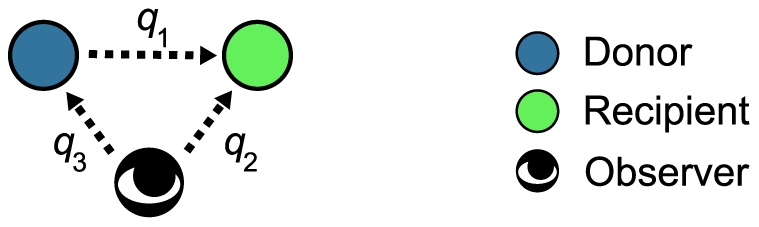
Three types of the probability 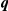. Three types of the probability 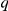 in a one-shot donation game. The donor knows the recipient's reputation with probability 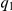. The observer knows the recipient's reputation and uses it to assign reputation to the donor with probability 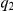. The observer observes the donor's action and assigns a new reputation to the donor with probability 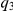.

In the donation game under a second-order social norm, we should distinguish between three types of the observation probability 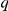, as shown in Fig. 7. 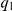 is the probability that the donor knows the recipient's reputation. 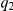 is the probability that the observer knows the recipient's reputation and uses it to assign a reputation to the donor. 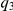 is the probability that the observer observes the donor's action and assigns a reputation to the donor. Observers are confined to a first-order social norm when 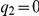 and can use complete second-order social norms when 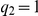.

If 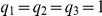, the discriminator is stable under three second-order social norms, i.e., simple standing, stern judging, and shunning [23]. Nowak & Sigmund [6], [7] studied the case 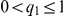 under image scoring (i.e., 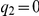). When 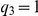, cooperation is difficult for a small value of 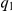 and a necessary condition for indirect reciprocity is given by 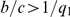 [7]. Although our model is different from theirs, our results are consistent with this necessary condition for their model. They also performed numerical simulations in which a player 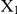 observes a game with probability 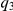 (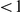) and updates the image score of the donor 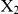 [6]. 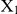 refers to the image score of 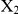 only when 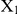 plays with 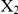 as donor. Panchanathan & Boyd [18] considered two different action rules, discriminator and contrite TFT, under a third-order standing norm. They found that both strategies can be ESS for 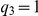. Brandt & Sigmund [21] numerically analyzed the case 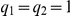 and 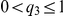. They showed that for a small 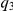, cooperation is relatively easily accomplished under image scoring and third-order standing than third-order judging. Following Mohtashemi & Mui [30], Brandt & Sigmund [31] investigated the image scoring (i.e., 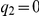) when 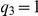 and 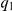 (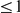) increases with time. They found that the trustful discriminator and the unconditional cooperator can stably coexist. Finally, Brandt & Sigmund [19] elaborated the case 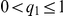, 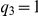 under image scoring (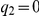) in various situations. Table 2 summarizes the previous models. In the present study, we conducted an exhaustive search of stable and cooperative pairs of social norms and action rules when 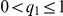, 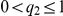, and 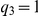.

**Table 2:** Comparison between different models.

Models	Parameter ranges		
Nowak & Sigmund [6]	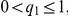	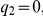	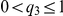
Nowak & Sigmund [7]	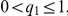	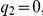	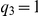
Panchanathan & Boyd [18]	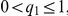	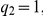	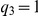
Brandt & Sigmund [21]	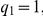	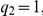	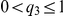
Mohtashemi & Mui [30]	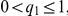	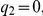	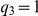
Brandt & Sigmund [31]	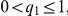	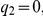	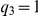
Brandt & Sigmund [19]	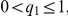	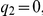	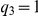
Our model	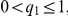	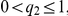	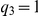

Comparison between observation probabilities in different models of indirect reciprocity. 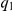 is the probability that the donor knows the recipient's reputation. 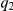 is the probability that the observer knows the recipient's reputation and uses it to assign a reputation to the donor. 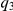 is the probability that the observer observes the donor's action and assigns a reputation to the donor. In most previous models, 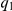 has been assumed to be real valued, whereas 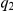 is binary, i.e., 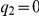 or 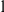. In our model, 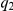 is also real valued such that observers as well as interacting players of the game have incomplete information about the recipient's reputation.

In the context of incomplete observation, most previous models of indirect reciprocity assumed that the ability of observers is either null (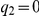) or complete (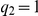) (see Table 2), which is in contrast with the graduated ability of observation (i.e., 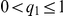) assumed for donors. If a player acts as a donor and an observer in different situations, it seems likely to assume real-valued 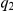 (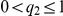). For this case, we showed that indirect reciprocity is possible for various values of 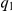 and 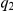.

Under incomplete observation, a small fraction of players may observe a donor 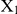
's action, and these observers may inform others of 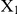
's reputation via gossip [10], [15]. Suppose that a player observes a one-shot game and propagates 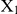
's reputation to the entire population with probability 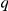 and that nobody observes the one-shot game with probability 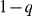. In this case, when 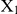 is selected as a recipient in a later one-shot game, the donor and the observer of this game may concomitantly know 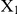
's reputation. Alternatively, suppose that the initial observers always exist and propagate 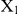
's reputation to a fraction, 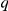, of players directly or indirectly. If 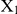 is later selected as recipient and the observer is always selected from the neighborhood of the donor in the social network of gossiping, it is probable that the donor and observer concomitantly know the recipient's reputation. Independent observation does not require these assumptions and may be more natural than concomitant observation. We showed that in our model, even with independent observation, cooperation is achieved in a large parameter region, albeit smaller than that for the concomitant observation.

Previous studies focused on the situation that donors, but not observers, have incomplete information about the society. Without an authority responsible for reputation assignment, we believe that donors and observers are temporary and not fixed roles for individuals such that observers as well as donors are exposed to incomplete information. The present results provide an important step toward understanding indirect reciprocity in self-sustaining societies.
